# Effectiveness, safety, and treatment patterns of GLP-1 receptor agonists in adolescents with obesity: a multicenter retrospective study from Saudi Arabia

**DOI:** 10.3389/fnut.2026.1902763

**Published:** 2026-07-27

**Authors:** Farah Kalagi, Lara Hamed, Sadeem Alrubaian, Fatimah Almuzain, Nisreen Alturkestany, Sulaiman Bah, Yasmin Algindan

**Affiliations:** 1 Department of Public Health, College of Public Health, Imam Abdulrahman Bin Faisal University, Dammam, Saudi Arabia; 2 Institutional Review Board (IRB), SMC Healthcare, Riyadh, Saudi Arabia; 3 Clinical Nutrition Department, Dr. Sulaiman Al Habib Hospital, Al-Khobar, Saudi Arabia; 4 Medical Allied Services Department, Imam Abdulrahman Bin Faisal University – King Fahd Hospital of the University, Dammam, Saudi Arabia; 5 Department of Clinical Nutrition, College of Applied Medical Sciences, Imam Abdulrahman Bin Faisal University, Dammam, Saudi Arabia

**Keywords:** adolescents, GLP-1 receptor agonists, Pediatric Obesity, Real-world evidence, Saudi Arabia, Treatment patterns, weight management

## Abstract

**Background:**

Real-world evidence on the use of glucagon-like peptide-1 (GLP-1) receptor agonists in pediatric obesity remains limited, particularly regarding treatment outcomes, safety, and clinical practice patterns.

**Objective:**

To evaluate changes in body weight and BMI z-score, assess safety outcomes, and describe treatment patterns among adolescents receiving GLP-1 receptor agonists in routine clinical practice in Saudi Arabia.

**Methods:**

A multicenter retrospective observational study was conducted using medical records of adolescents aged 12–18 years who received GLP-1 receptor agonists between January 2021 and December 2024. Data on demographic and clinical characteristics, treatment patterns, and outcomes were collected. Within-group changes were assessed using the Wilcoxon signed-rank test, and between-group comparisons were performed using the Kruskal–Wallis test.

**Results:**

A total of 81 patients were included. GLP-1 therapy was associated with modest reductions in weight and BMI z-score across treatment groups, depending on the GLP-1 receptor agonist used, with no statistically significant differences between groups. Median percentage weight reduction ranged from −2 to −7%. Adverse events were infrequently documented and, when reported, predominantly gastrointestinal. Endocrinologists issued most prescriptions; the median treatment duration was 4 months, dose titration was documented in 37% of patients, and referral for bariatric surgery occurred in 9% of cases.

**Conclusion:**

In routine clinical practice, GLP-1 receptor agonists were associated with modest weight reduction and were generally well tolerated among adolescents with obesity. Variability in treatment patterns highlights the need for more standardized approaches to optimize the use of GLP-1 therapies in pediatric obesity management.

## 1 Introduction

Childhood and adolescent obesity continue to represent a major global health concern, with rising prevalence and significant long-term metabolic and psychological consequences (1). Recent global estimates also indicate a sustained rise in childhood and adolescent obesity, with further increases expected by 2050 (2). Lifestyle interventions remain the cornerstone of obesity management and have demonstrated beneficial effects on weight-related outcomes in children and adolescents with obesity (3, 4). However, achieving sustained weight reduction in routine clinical practice remains challenging, particularly among adolescents, due to difficulties with long-term adherence and maintaining behavioral changes (5).

In response, some clinical guidelines support the use of pharmacological therapies in selected patients with obesity who do not respond adequately to behavioral interventions (6). Among these therapies, glucagon-like peptide-1 (GLP-1) receptor agonists have gained prominence following randomized controlled trial evidence demonstrating their effectiveness in reducing body weight and body mass index in adults. Subsequent studies have confirmed similar benefits in adolescents (7, 8). Recent evidence further supports the use of GLP-1 receptor agonists (GLP-1 RAs) in adolescents with obesity, demonstrating their effectiveness and acceptable safety profile in this population. (9, 10) However, the translation of these findings into routine clinical practice remains inconsistent. Although recent systematic reviews support the effectiveness of GLP-1 receptor agonists in children and adolescents with obesity, their use remains accompanied by safety and implementation concerns, particularly regarding gastrointestinal adverse events, treatment adherence, and the limited availability of long-term pediatric data (9, 11, 12).

Recent national data from the United States indicate that the use of obesity pharmacotherapy among adolescents remains very limited despite increasing availability, with only 0.5% of adolescents with obesity receiving a medication prescription in 2023. Most prescriptions were observed among individuals with severe obesity (13). Furthermore, even within structured clinical programs, pharmacological treatment is not uniformly implemented, reflecting substantial variability in prescribing practices (9). This variability highlights the complexity of clinical decision-making, where factors beyond clinical efficacy alone influence treatment initiation, dose titration, continuation, and discontinuation. These include patient characteristics, treatment adherence, clinician experience, and healthcare system-related constraints, such as medication cost, insurance coverage, availability, and concerns regarding long-term safety (9, 14, 15).

In Saudi Arabia, national guidance for obesity management emphasizes lifestyle modification as the first-line approach, while recognizing the role of pharmacological therapy in selected cases (16). However, despite the availability of general clinical recommendations for obesity management, evidence describing the use of GLP-1 receptor agonists in pediatric populations remains limited (6, 16, 17). In addition, emerging studies suggest variability in prescribing practices and clinical use across routine care settings. In particular, patterns related to treatment initiation, dose titration, switching, and discontinuation remain insufficiently characterized (9, 15).

Studies in adolescents have also reported variability in treatment response, adherence, follow-up, and continuation of therapy, highlighting the complexity of implementing GLP-1 therapy in clinical practice (17, 18). Understanding these aspects is essential to bridge the gap between clinical recommendations and actual clinical practice and to optimize the use of pharmacological therapies in pediatric obesity management.

Therefore, this study aimed to evaluate the effectiveness, safety, and treatment patterns of GLP-1 receptor agonists among adolescents with obesity in Saudi Arabia.

## 2 Materials and methods

### 2.1 Study design and setting

This study employed a retrospective observational design based on medical record review. It included adolescents aged 12–18 years who received glucagon-like peptide-1 receptor agonist (GLP-1 RA) therapy for obesity management between January 2021 and December 2024. Data were collected from both government and private healthcare institutions across different regions of Saudi Arabia, including Riyadh and the Eastern Province (Dammam and Al Khobar).

**Table:** 

Hospital	Type	Location
King Fahad Medical City	Governmental	Riyadh
King Fahd Hospital of the University	Governmental	Dammam
Dr. Sulaiman Al Habib Hospital	Private	Al Khobar
Specialized Medical Center	Private	Riyadh

Multiple centers from different healthcare sectors and geographic regions were included to enhance sample representativeness and capture variability in prescribing practices and clinical management across routine care settings.

The retrospective design enabled the assessment of clinical practice, including routine prescribing patterns, patient characteristics, and documented clinical outcomes. No direct patient contact occurred, and all data were obtained exclusively from existing medical records.

### 2.2 Study population

The study population comprised adolescents aged 12–18 years diagnosed with obesity and treated with GLP-1 receptor agonists during the study period.

Eligible cases were identified through a review of electronic medical records from participating healthcare institutions. Patients were included if they:

1. Were aged 12–18 years at treatment initiation,
2. Had a documented diagnosis of obesity, and
3. Received at least one documented prescription of a GLP-1 and initiated treatment.

Patients were excluded if medical records were incomplete to the extent that key study variables could not be retrieved. Although the study targeted adolescents aged 12–18 years, two patients who initiated GLP-1 therapy at ages 10–11 years were retained to reflect real-world clinical practice and off-label use.

### 2.3 Data collection

Data were retrospectively extracted manually from electronic medical records and entered into a standardized data collection form developed for this study. Data collection was conducted by the principal investigator with assistance from co-investigators across participating institutions.

Co-investigators received standardized training from the principal investigator before data collection. Training included a detailed explanation of the data abstraction form, variable definitions, and procedures for medical record review. Training sessions were conducted individually via online meetings, and co-investigators were encouraged to contact the principal investigator for clarification as needed during data collection.

Collected variables included demographic characteristics (age, sex, hospital type, and region), baseline clinical measures (weight, height, and BMI), and treatment-related variables (type of GLP-1 receptor agonist, starting dose, dose titration, treatment duration, treatment delay, and baseline laboratory investigations). Follow-up data included the last recorded weight and BMI.

Additional variables included documented adverse events, comorbid medical conditions, concomitant medications, and, when available, lifestyle counseling.

### 2.4 Study variables and outcomes

The study outcomes were defined in alignment with the study objectives, including effectiveness, safety, and treatment patterns.

To assess effectiveness, the primary outcome was the change in body weight from baseline to the last available follow-up. Percentage weight change was calculated as [(follow-up weight−baseline weight)/baseline weight] × 100, then categorized into clinically meaningful groups.

BMI z-score was evaluated as a secondary measure of treatment response to account for age- and sex-related growth variation inherent to adolescence. BMI z-scores were calculated using the World Health Organization (WHO) 2007 growth reference for children and adolescents aged 5–19 years, applying the LMS method, which adjusts for the skewness (L), median (M), and coefficient of variation (S) of the BMI distribution at each age and sex stratum.

To assess safety, documented adverse events during treatment were recorded.

To evaluate treatment patterns, variables related to clinical use were examined, including dose titration, treatment duration, treatment delay, and discontinuation or modification of therapy.

Independent variables included demographic characteristics, baseline clinical measures, and treatment-related variables. Additional variables included baseline laboratory investigations, comorbid conditions, concomitant medications, and referral for bariatric surgery.

### 2.5 Sample size considerations

All medical records that met the inclusion and exclusion criteria during the study period were included. A total of 107 medical records were reviewed; 81 patients met the eligibility criteria and were included in the final analysis. To contextualize sample adequacy, a theoretical sample size was estimated using a paired mean-difference approach, assuming a conservative expected weight change of 3 kg, a standard deviation of paired weight differences of approximately 11.9 kg, a two-sided *α* of 0.05, and 80% power; the estimated minimum sample size was approximately 124 patients. Based on the achieved sample size of 81 patients, the study had 80% power to detect a mean weight change of at least 3.7 kg. Accordingly, the sample was considered acceptable for descriptive analyses and for detecting moderate within-patient changes in body weight.

### 2.6 Statistical methods

Statistical analyses were conducted using SPSS version 26 (IBM Corp., Armonk, NY, United States). Missing data accounted for 12% of the study dataset. To minimize potential bias and preserve statistical power, missing values in the treatment-end weight and treatment-end height variables were handled using multiple imputations by chained equations (MICE). Predictive mean matching (PMM) was used to impute these continuous outcomes, generating 20 imputed datasets with five iterations per imputation (default setting). The imputation model included baseline weight, height, body mass index, age at treatment initiation, sex, region, treatment duration, starting dose, and last dose as predictors. To avoid circular prediction, treatment-end weight and end-height were not permitted to predict one another, and all predictor variables were retained as observed (i.e., not imputed). The first completed dataset was used for subsequent analyses. A sensitivity analysis was performed by repeating the primary analyses using the complete-case dataset and comparing the results with those obtained from the imputed dataset. Similar estimates and conclusions across the two analyses were considered evidence that the findings were robust to the handling of missing outcome data.

Continuous variables were assessed for normality and presented as medians and interquartile ranges (IQR), while categorical variables were summarized as frequencies and percentages.

Within-group changes in weight and BMI z-score were assessed using the Wilcoxon signed-rank test. Between-group comparisons were conducted using the Kruskal–Wallis test. Associations between categorical variables were examined using the Chi-square test or Fisher’s exact test, as appropriate. A two-tailed *p*-value <0.05 was considered statistically significant. Given the exploratory nature of the analyses, results were interpreted cautiously.

### 2.7 Ethical considerations

Ethical approval was obtained from the Institutional Review Board of Imam Abdulrahman bin Faisal University (IRB-PGS-2024-03-742), King Fahad Medical City (IRB No. 24-652E), Dr. Sulaiman Al Habib Medical Group (IRB No. HAP-01-R-082), and the Specialized Medical Center (IRB No. 001–2025) before data collection. As this was a retrospective study based solely on medical record review, no direct patient contact occurred, and informed consent was waived.

All data were handled confidentially and anonymized at extraction. Each patient was assigned a unique study identifier, and no personal identifiers were retained. To protect institutional confidentiality, participating hospitals were coded as “governmental” or “private.” Access to records was restricted to the research team, and data were stored in secure, password-protected files. The study posed no additional risk to patients.

## 3 Results

### 3.1 Participant and baseline clinical characteristics

A total of 81 adolescents treated with GLP-1 receptor agonists were included. Most participants were from private hospitals (79%) and the Eastern Province (Dammam and Al Khobar) (56%), with the remainder from government hospitals (21%) and Riyadh (44%). The sample was nearly evenly split by sex (51% female, 49% male). The median age at treatment initiation was 16 years (IQR: 14–17), and 51% had at least one documented comorbidity at baseline (Table 1).

**Table 1:** Baseline demographic and clinical characteristics of the study participants.

Characteristic		Overall (*n* = 81)
		*n* (%)
Region	Riyadh	36 (44%)
	Dammam/Al-Khobar	45 (56%)
Hospital type	Government	17 (21%)
	Private	64 (79%)
Age group	Adolescent	81 (100%)
Age at start of treatment (years) Median (Q1–Q3)		16 (14–17)
Gender	Male	40 (49%)
	Female	41 (51%)
Presence of comorbidity	Yes	41 (51%)
	No	40 (49%)

### 3.2 Distribution of comorbidities

**Figure 1:**
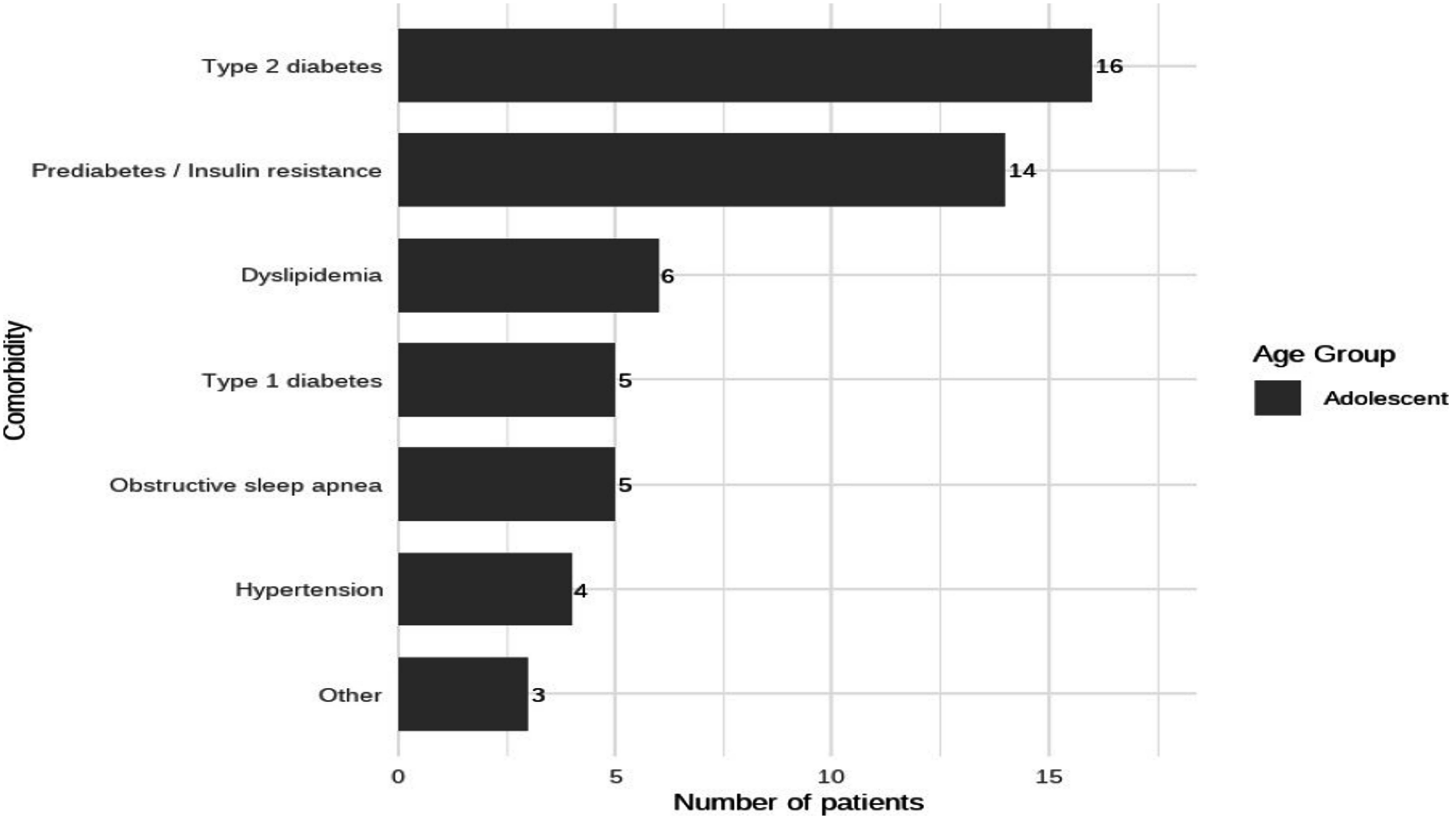
Distribution of comorbidities in the Cohort (*n* = 81).

Among participants with documented comorbidities (*n* = 41), type 2 diabetes mellitus was the most frequently recorded condition (*n* = 16, 39%), followed by insulin resistance (*n* = 14, 34%). Less frequent comorbidities included dyslipidemia (*n* = 6, 15%), type 1 diabetes and obstructive sleep apnea (*n* = 5 each, 12%), hypertension (*n* = 4, 10%), and other comorbidities (*n* = 3, 7%) (Figure 1).

### 3.3 Weight and BMI z-score outcomes

Significant reductions were observed within several treatment groups: semaglutide and tirzepatide produced meaningful decreases in body weight (−3.0%, *p* < 0.001 and −7.3%, *p* = 0.010, respectively), while BMI z-score declined significantly with semaglutide (*Δ* − 0.89, p < 0.001), liraglutide (Δ − 0.60, *p* = 0.034), and tirzepatide (Δ − 2.14, *p* = 0.018). In contrast, weight reductions with liraglutide (−1.8%, *p* = 0.113) and dulaglutide (−2.9%, *p* = 0.281), and the BMI z-score change with dulaglutide (Δ − 1.05, *p* = 0.062), were not statistically significant. Despite these within-group improvements, no statistically significant differences were detected between treatment groups (Kruskal–Wallis *p* > 0.05 for all comparisons) (Table 2).

**Table 2:** Weight and BMI z-score outcomes by GLP-1 receptor agonist type.

GLP-1 type	*n*	Weight (kg)				BMI z-score			
		Baseline median (IQR)	Final median (IQR)	Δ (%) median (IQR)	Within-group *p*-value^b^	Baseline median (IQR)	Final median (IQR)	Δ median (IQR)	Within-group *p*-value^b^
Semaglutide	36	109.0 (88.6–127.2)	96.5 (75.0–123.5)	−3.0% (−10.1 to 0)	**<0.001**	11.79 (7.61–16.42)	9.72 (4.65–13.81)	−0.89 (−3.52 to −0.03)	**<0.001**
Liraglutide	24	100.4 (88.2–120.2)	95.0 (79.0–123.6)	−1.8% (−13.0 to 2.4)	0.113	12.25 (9.24–16.19)	10.83 (7.75–13.50)	−0.60 (−4.23 to 0.52)	**0.034**
Tirzepatide	15	96.0 (80.5–112.0)	91.8 (75.5–97.5)	−7.3% (−13.0 to 0)	**0.010**	10.94 (8.58–14.27)	9.51 (6.44–11.04)	−2.14 (−3.23 to 0.06)	**0.018**
Dulaglutide	6	109.0 (87.2–127.0)	100.0 (78.2–130.8)	−2.9% (−7.1 to −0.6)	0.281	10.92 (8.73–13.98)	9.47 (7.43–13.84)	−1.05 (−2.01 to −0.81)	0.062
Between-group *p*-value^a^		0.672	0.659	0.937	—	0.869	0.637	0.974	—

Values are presented as median (interquartile range). BMI z-scores were calculated using the WHO 2007 growth reference (ages 5–19 years) via the LMS method with extended correction for values exceeding ±3 SD, applied to BMI recomputed from measured weight and height.

Bold values indicate statistical significance (*p* < 0.05).

a Kruskal–Wallis test across GLP-1 treatment groups.

b Wilcoxon signed-rank test comparing baseline to final values within each GLP-1 group.

IQR, interquartile range; Δ, change from baseline to final assessment. Results should be interpreted cautiously due to small subgroup sizes, particularly for dulaglutide (*n* = 6).

### 3.4 Weight change categories by GLP-1 type

The distribution of weight change categories varied across GLP-1 treatment groups (Table 3). Significant weight reduction of at least 10% was achieved by approximately one-third of participants in the liraglutide (8, 33%) and tirzepatide (5, 34%) groups, followed by semaglutide users (10, 28%) and the dulaglutide group (1, 17%). Moderate weight loss (5 to <10%) was most common among tirzepatide (3, 20%) and semaglutide users (7, 19%).

**Table 3:** Weight Change categories by GLP-1 type.

Type of GLP-1*	Semaglutide		Liraglutide		Tirzepatide		Dulaglutide	
	*n* = 36		*n* = 24		*n* = 15		*n* = 6	
Weight change category	*n*	%	*n*	%	*n*	%	*n*	%
≥10% loss	10	28%	8	33%	5	34%	1	17%
5–<10% loss	7	19%	3	13%	3	20%	1	17%
<5% loss	7	19%	5	21%	2	13%	2	33%
No change	6	17%	0	0%	3	20%	1	17%
Weight gain	6	17%	8	33%	2	13%	1	17%

Percentages are calculated within each GLP-1 type. Results are descriptive and should be interpreted cautiously due to small subgroup sizes.

^*^GLP-1 types are based on treatment at baseline.

### 3.5 Safety outcomes

Overall, most patients did not have documented adverse events during GLP-1 therapy (74%). When present, adverse events were predominantly gastrointestinal, involving both upper and lower GI symptoms. The highest proportion of reported adverse events was observed among liraglutide users (38%), followed by dulaglutide (33%), semaglutide (22%), and tirzepatide (13%). No severe or life-threatening adverse events were documented. However, the severity of adverse events and their direct impact on treatment continuation were not consistently recorded in the medical records, limiting further assessment of their clinical impact.

### 3.6 Treatment setting and prescribing characteristics

Treatment patterns, including prescribing specialties and clinical management practices, showed that most prescriptions were issued by endocrinologists (74%), followed by obesity/bariatric surgeons (14%) and family medicine/internal medicine/nephrology physicians (12%).

The median treatment duration was 4 months (IQR: 3–8). Treatment initiation was delayed in 17% of patients, with a median delay of 6 months (IQR: 4.2–11). Changes in GLP-1 therapy were uncommon (5%), and all switches were to semaglutide. Dose titration was documented in 37% of patients. Lifestyle counseling was provided to 63% of patients, while referrals for bariatric surgery were relatively infrequent (9%) (Table 4).

**Table 4:** Prescribing characteristics and clinical management patterns.

Characteristic		Overall (*n* = 81)	
		*n*	%
Prescriber specialty	Endocrinology (Adult/Pediatric)	60	74%
	Family medicine/internal medicine/nephrology	10	12%
	Obesity/bariatric/surgery	11	14%
Treatment duration (months) median (Q1–Q3)		4 (3–8)	
Treatment delay^*^	Yes	14	17%
	No	67	83%
Treatment delay (months)		6 (4.2–11)	
GLP-1 change	Yes	4	5%
	No	77	95%
GLP-1 changed to	Semaglutide	4	100%
Dose titration	Yes	30	37%
	No	51	63%
Lifestyle advice given	Yes	51	63%
	No	30	37%
Referred for bariatric surgery	Yes	7	9%
	No	74	91%

^*^Delays in treatment initiation despite eligibility based on age and BMI criteria.

### 3.7 Treatment patterns and concomitant medications

Baseline dosing patterns varied among GLP-1 receptor agonists. Semaglutide was initiated at a median dose of 1.00 mg (IQR: 0.25–2.50) and titrated to 1.35 mg (IQR: 0.50–6.00), while liraglutide increased from 0.50 mg (IQR: 0.25–0.80) to 2.70 mg (IQR: 0.75–3.00). Tirzepatide also showed an increase in doses over time, whereas dulaglutide dosing remained stable (Table 5).

**Table 5:** Dosing patterns by GLP-1 type Median (Q1–Q3). Comparisons across GLP-1 types are exploratory in nature due to small subgroup sizes.

Dose	Semaglutide	Liraglutide	Tirzepatide	Dulaglutide
	*n* = 36	*n* = 24	*n* = 15	*n* = 6
Starting dose (mg)	1.00 (0.25–2.50)	0.50 (0.25–0.80)	0.25 (0.25–2.50)	1.50 (1.50–1.50)
Last dose (mg)	1.35 (0.50–6.00)	2.70 (0.75–3.00)	1.00 (1.00–7.50)	1.50 (1.50–1.50)

Across GLP-1 groups, concomitant medication varied substantially. The proportion of patients receiving additional therapies was highest with dulaglutide (5/6, 83%), followed by liraglutide (11/24, 46%), tirzepatide (9/15, 60%), and semaglutide (8/36, 22%). Among those on concomitant medications, metformin predominated across all groups: 6/8 (75%) in the semaglutide group, 10/11 (91%) in the liraglutide group, 4/4 (100%) in the tirzepatide group, and 3/5 (60%) in the dulaglutide group. Insulin use was infrequent overall but relatively higher in the dulaglutide group (2/5, 40%), as shown in Table 6.

**Table 6:** Concomitant medications by GLP-1 type.

Type of GLP-1		Semaglutide		Liraglutide		Tirzepatide		Dulaglutide	
		*n* = 36		*n* = 24		*n* = 15		*n* = 6	
		*n*	%	*n*	%	*n*	%	*n*	%
On other medications	Yes	8	22%	13	54%	9	60%	5	83%
	No	24	67%	11	46%	4	27%	0	0%
	Not assessed	4	11%	0	0%	2	13%	1	17%
Type of other medications	Glucagon	1	12.5%	0	0%	0	0%	0	0%
	Insulin	1	12.5%	0	0%	0	0%	2	40%
	Metformin	6	75%	10	77%	4	44%	3	60%

Patients may have received more than one concomitant medication, and only selected medication categories are presented.

Not all concomitant medications are presented.

### 3.8 Prescriber specialty and clinical practice variations

By specialty, no statistically significant differences were observed in treatment delays, delay duration, treatment duration, or GLP-1 selection. However, baseline laboratory testing differed significantly across specialties (*p* < 0.001), with substantially lower testing rates in bariatric surgery than in endocrinology and family/internal medicine (Table 7). These findings should be interpreted cautiously due to small subgroup sizes.

**Table 7:** Crosstabulation of physicians’ specialty across key clinical variables.

Prescriber specialty	Endocrinology (adult/pediatric)		Family medicine/internal medicine/nephrology		Bariatric surgery		*p*-value^2^
	*n* = 60		*n* = 10		*n* = 11		
	*n*	%	*n*	%	*n*	%	
Treatment delayed (yes)^*^	11	20%	1	10%	1	9%	0.701
Time of delay (months) Median (Q1–Q3)	5 (4.0–9.8)		N/A	N/A	8 (8.0–8.0)		0.202
Baseline labs done (yes)	52	88%	10	100%	4	36%	**<0.001**
GLP-1 Type							
*Semaglutide*	26	43%	6	60%	4	36%	0.682
*Liraglutide*	19	32%	2	20%	3	27%	
*Tirzepatide*	10	17%	1	10%	4	36%	
*Dulaglutide*	5	8%	1	10%	0	0%	
Treatment duration (months) ^**^Median (Q1–Q3)	5 (3–8)		3 (3–4)		3 (3–5)		0.337

*p*-values for categorical variables were derived using Chi-square or Fisher’s exact tests, as appropriate, and Kruskal–Wallis tests were used for continuous variables.

Patients with treatment duration <3 Months were excluded from this analysis.

*Delay in treatment initiation despite eligibility based on age and BMI criteria.

**Other includes nephrology.

Results should be interpreted cautiously due to small subgroup sizes.

## 4 Discussion

This retrospective study provides insights into the effectiveness, safety, and clinical use of GLP-1 among adolescents with obesity in Saudi Arabia. Overall, GLP-1 therapy showed modest reductions in body weight and BMI z-score, with noticeable variability across treatment types and among patients. These findings are generally consistent with previous clinical trials and observational studies, which have shown that GLP-1 can lead to meaningful weight reduction in adolescents (8, 10, 15, 17, 18). However, the magnitude of weight loss in the current study was somewhat lower than that reported in controlled trials, reflecting differences in adherence, treatment duration, and clinical practice.

Weight and BMI z-score reductions were observed across all GLP-1 agents, with no statistically significant differences between treatments. Similar findings have been reported in previous studies demonstrating consistent weight reduction with GLP-1 (19). Evidence from observational studies in adolescents supports these findings, although the magnitude varies. A small cohort of adolescents treated with liraglutide (*n* = 22) showed a significant reduction in BMI-SDS from +2.63 to +2.40 over a mean treatment duration of 8.2 months (17). In a larger study of 966 adolescents, the mean BMI reduction was −2% overall, reaching −10% among patients with higher adherence (≥7 prescriptions) (18).

Randomized controlled trials have reported greater weight reduction under controlled conditions. The SURMOUNT-1 trial (20) included 2,539 adults with obesity and showed mean weight reductions of −15.0 to −20.9% with tirzepatide, compared with −3.1% with placebo over 72 weeks. Similarly, the STEP TEENS trial (8) included 201 adolescents and demonstrated a mean BMI reduction of −16.1% with semaglutide, compared with a 0.6% increase with placebo after 68 weeks. In the current study, tirzepatide demonstrated slightly greater reductions in body weight and BMI z-score than the other GLP-1 receptor agonists; however, these differences were not statistically significant. Similar findings have been reported in recent real-world studies, in which treatment responses were heterogeneous and no independent association between treatment group and clinically meaningful weight loss was observed after adjustment for baseline characteristics (21).

Adverse events were infrequently documented; most patients reported none. When present, they were predominantly gastrointestinal, with no severe events observed, and hypoglycemia was rare. These findings are consistent with previous evidence showing an increased incidence of gastrointestinal adverse events without a significant rise in serious events among adolescents (9). Similar patterns have been reported in larger pooled analyses, where gastrointestinal adverse events were more frequent with GLP-1 therapies, with no significant increase in hepatic or pancreatic complications (22). Broader reviews have also reported mixed findings regarding rare but potentially serious adverse events (23).

Treatment patterns showed that most GLP-1 prescriptions were issued by endocrinologists, with limited involvement from other specialties, consistent with previous studies indicating that specialists primarily manage obesity pharmacotherapy (14, 24). Treatment duration was relatively short (median 4 months), and dose titration was documented in a minority of patients. Similar patterns in treatment duration, dose adjustment, and adherence have been reported in previous studies, reflecting differences in clinical practice and follow-up (15, 18). Lifestyle counseling was documented in approximately two-thirds of patients, indicating that pharmacological treatment was often combined with non-pharmacological approaches, as recommended in clinical guidelines (6). In contrast, treatment switching was uncommon.

Referral for bariatric surgery was reported in 9% of patients (7/81), indicating that a small proportion of this cohort was referred to surgery. Bariatric surgery is recommended in selected adolescents with severe obesity within a comprehensive management approach. (6)

Dosing patterns showed escalation for semaglutide, liraglutide, and tirzepatide, while dulaglutide remained stable. For example, semaglutide increased from 1.00 to 1.35 mg, and liraglutide from 0.50 to 2.70 mg, consistent with the previous report (9). Concomitant medication use was more frequent with dulaglutide (83%), tirzepatide (60%), and liraglutide (54%) than with semaglutide (22%), with metformin being the most commonly prescribed co-therapy (6).

No statistically significant differences were observed across prescriber specialties in treatment delay, duration, or GLP-1 selection. However, baseline laboratory testing differed significantly, with lower rates among patients undergoing bariatric surgery than among those in endocrinology and family/internal medicine. Clinical guidelines emphasize the importance of comprehensive assessment, including evaluation of obesity-related comorbidities (10, 16, 25, 26). Variations in clinical practice among physicians have also been reported (14).

### 4.1 Strengths and limitations

This study provides insights into the use of GLP-1 receptor agonists among adolescents in Saudi Arabia, a population for which available data are limited. The inclusion of both government and private healthcare settings enhances representativeness, while the use of routinely collected clinical data reflects actual prescribing practices. The study also provides a detailed evaluation of treatment patterns and clinical outcomes.

Limitations are primarily related to the use of medical records, including variability in documentation and incomplete data capture. Some variables, such as adverse event severity and lifestyle adherence, were not consistently recorded, which may have affected the accuracy of outcomes. Although data abstractors received standardized training, formal inter-rater reliability testing (e.g., Cohen’s kappa) was not performed. Therefore, some variability in data abstraction across sites cannot be excluded. Missing outcome data were addressed using multiple imputations by chained equations (MICE). In addition, data collection across the participating centers required approximately 1 year. Given the practical time constraints of the study, it was not feasible to extend the data collection period further to achieve the theoretical sample size. The relatively small sample sizes within treatment subgroups may have reduced the statistical power to detect differences between individual GLP-1 receptor agonists. Therefore, the absence of statistically significant differences between treatment groups should be interpreted with caution, as it may reflect limited statistical power rather than true differences between treatments.

Larger multicenter studies with greater statistical power are needed to enable more robust comparisons between individual GLP-1 receptor agonists. In addition, more standardized data collection across healthcare settings would improve the completeness and consistency of routinely collected clinical data.

## 5 Conclusion

GLP-1 receptor agonists were associated with modest reductions in weight and BMI z-score, with no significant differences between treatments. The therapies were generally well tolerated, with few reported adverse events. Variability in prescribing practices highlights the need for more standardized approaches to optimize treatment use.

## Data Availability

The datasets generated and/or analyzed during the current study are not publicly available due to privacy and ethical restrictions. Requests to access the datasets should be directed to the corresponding author and will be considered in accordance with institutional policies, ethical requirements, and applicable approvals.
