# Supplementary material for: Effectiveness, safety, and treatment patterns of GLP-1 receptor agonists in adolescents with obesity: a multicenter retrospective study from Saudi Arabia
Source: Front Nutr. 2026 Jul 27;13:1902763. doi: 10.3389/fnut.2026.1902763 (PMC13455499; doi:10.3389/fnut.2026.1902763)
Supplement: Supplementary file 1 [file Table_1.docx]

Supplementary Material

# 1 Supplementary Data

**Supplementary Table 1.** Standardized Data Abstraction Form and Variable Definitions

| Domain | Variable Extracted | Description / Coding |
| --- | --- | --- |
| Patient Demographics | Patient ID | Unique anonymized identifier |
|  | Age at treatment start (years) | Continuous |
|  | Gender | Male / Female |
|  | Region | Dammam / Riyadh / AlKhobar |
| Clinical Characteristics | Comorbidities | Free text; later categorized |
|  | Comorbidity type | T1DM, T2DM, Prediabetes, Dyslipidemia, OSA, Hypertension, PCOS, Insulin resistance, NAFLD, etc. |
|  | Baseline labs done | Yes / No |
|  | Lab details | CBC, HbA1c, Lipid profile, TSH, T4, Renal panel, Liver panel, etc. |
| Medication History | Home medication | Yes / No |
|  | Medication name | Metformin, insulin, glucagon, etc. |
|  | Prescriber specialty | Endocrinology (Adult/Pediatric), Family Medicine, Bariatric Surgery, General Pediatrics, Clinical Nutrition |
| Lifestyle & Counseling | Lifestyle advice given | Yes / No |
|  | Referred to bariatric surgery | Yes / No |
| Treatment Details | Type of GLP‑1 used | Semaglutide, Liraglutide, Dulaglutide, Tirzepatide |
|  | Starting dose | mg or mL |
|  | Last dose | mg or mL |
|  | Dose titration | Yes / No |
|  | Medication change | Yes / No |
|  | Changed to | New GLP‑1 agent, if applicable |
|  | Treatment delayed | Yes / No |
|  | Delay reason | BMI, labs, clinical factors |
|  | Delay duration (months) | Numeric |
| Anthropometrics | Baseline weight (kg) | Continuous |
|  | Baseline height (cm) | Continuous |
|  | Baseline BMI | Continuous |
|  | End weight (kg) | Continuous |
|  | End height (cm) | Continuous |
|  | End BMI | Continuous |
|  | Weight change (kg) | Continuous (gain or loss) |
| Adverse Events | Documented adverse events | Nausea, vomiting, constipation, diarrhea, abdominal pain, hypoglycemia, none |
|  | Adverse event category | GI upper/lower, hypoglycemia, not assessed |
| Treatment Duration | Duration of treatment (months) | Continuous |
| Outcome Variables | BMI change | Continuous |
|  | Weight change category | Loss / Gain / No change |
|  | Treatment response | Based on BMI or weight change |

Several variables were documented as free-text entries in the medical records and were subsequently grouped into analytical categories.
